# Dual learning systems in talker identification: the effects of language, accent, and feedback

**DOI:** 10.3758/s13414-025-03201-8

**Published:** 2025-12-25

**Authors:** Shengyue Xiong, Zhe-chen Guo, Casey L. Roark, Gangyi Feng, Bharath Chandrasekaran

**Affiliations:** 1 Department of Communication Sciences and Disorders, Northwestern University, 2240 Campus Drive, Evanston, IL 60208 USA; 2 Department of Psychology, University of New Hampshire, Durham, NH 03824 USA; 3 Department of Linguistics and Modern Languages, Brain and Mind Institute, The Chinese University of Hong Kong, Leung Kau Kui Building, Shatin, N.T., Hong Kong SAR, China; 4 Brain and Mind Institute, The Chinese University of Hong Kong, Shatin, N.T., Hong Kong SAR, China

**Keywords:** Talker identification, Dual-learning systems, Language familiarity effect, Accented speech, Drift diffusion modeling

## Abstract

Talker identification categorizes variable speech signals into stable talker representations, a process facilitated by language and accent familiarity. The dual learning systems (DLS) model posits that speech category learning involves a “reflective” system based on explicit rules and a “reflexive” system based on stimulus-reward associations, with reflexive learning dominating in later stages. In this study, we leverage the DLS framework to investigate talker learning by training Mandarin-speaking listeners to identify talkers in native (Mandarin) and nonnative languages with native (English) or nonnative, but familiar accents (Mandarin-accented English) contexts. Listeners received either using full (e.g., *Incorrect. It’s Talker 1*) or minimally informative (e.g., *Incorrect*) feedback, encouraging reflective or reflexive learning, respectively. We assessed identification performance through accuracy and response times and analyzed the underlying decision processes using drift diffusion models. Results showed that language and accent familiarity improved accuracy and response times. At later training stages, minimal feedback, which promotes reflexive learning according to the DLS model, facilitated faster identification and more efficient decision-making, particularly in the nonnative language context (English). The findings highlight the benefit of reflexive learning in talker identification through improved response efficiency and the need to consider decision dynamics in this process. The data, materials, and analysis code are available online (https://osf.io/g7r9q/).

## Introduction

Learning to identify a talker is an important ability that guides and enhances social interactions. This ability requires mapping continuous and variable spoken signals onto a meaningful and stable representation of a talker—a many-to-one categorization process that integrates multiple sources of acoustic-phonetic, lexical-semantic, and indexical information. Over the last decade, there has been substantial work focusing on speech perception, primarily using phonetic contrasts, through the lens of categorization (Chandrasekaran, Koslov et al., 2014a; Chandrasekaran, Yi et al., 2014b; Holt & Lotto, 2010; Maddox et al., 2013; Roark et al., 2024; Samuel & Kraljic, 2009). The extent to which a similar approach could be leveraged for talker categorization is unclear and is the focus of the current study. Specifically, can our current understanding of speech category learning be applied to promote learning new talker voices, particularly those speaking an unfamiliar language?

Over the past few decades, a number of theories have been proposed to explain how people acquire novel categories (for a review, see Minda et al., 2024). Extensive neuropsychological, neuroimaging, and behavioral evidence has demonstrated that category learning involves multiple systems (Ashby & Maddox, 2005; Knowlton, 1999; Nomura & Reber, 2008; Poldrack & Packard, 2003), with the COmpetition between Verbal and Implicit Systems (COVIS) model (Ashby et al., 1998) providing a robust theoretical scaffolding for the multiplicity of learning systems in the visual domain. The COVIS model suggests two functionally and neurally dissociable learning systems: a “reflective” system that uses executive attention and working memory to test explicit categorization rules on the basis of informational feedback, and a “reflexive” procedure-based system that relies less on executive attention and working memory and implicitly associates perceptions with actions that are reinforced by feedback. These dual systems are complementary but one may be more dominant than the other depending on factors such as the structure of the categories, the extent of feedback available to the learner, and the stage of learning. Specifically, rule-based categories engage explicit learning, while information-integration categories rely on the implicit system (Ashby et al., 1998). Full feedback benefits learning categories with verbalizable rules, while minimal feedback is better for complex categories that integrate multiple dimensions (Maddox et al., 2008; Yi & Chandrasekaran, 2016). Early learning is primarily mediated by reflective systems, while later learning shifts to more reflexive, procedural-based systems (Ashby & Maddox, 2011; Chandrasekaran, Koslov et al., 2014a; Chandrasekaran, Yi et al., 2014b).

More recently, the COVIS model has been extended into the auditory domain, with the concept of dual-learning systems (DLS) being applied to examine nonnative speech categories and how the learning of these categories can be facilitated through manipulation of feedback (Chandrasekaran, Koslov et al., 2014a; Chandrasekaran, Yi et al., 2014b, Chandrasekaran et al., 2016; Gabay et al., 2023; Roark & Holt, 2018; Yi & Chandrasekaran, 2016). An illustrative study is Chandrasekaran, Koslov et al. (2014a), Chandrasekaran, Yi et al. (2014b), where native^1^English speakers were trained to identify Mandarin (nonnative) lexical tones while receiving either fully informative feedback providing detailed and comprehensive responses (e.g., *Incorrect. The answer is category 1*) or minimally informative feedback regarding response correctness (e.g., *Correct* or *Incorrect*). These two types of feedback are of theoretical interest as they engage different learning systems under the COVIS/DLS framework. Full feedback promotes the generation and testing of explicit rules critical to the reflective system, but hinders the transfer of control to the reflexive system by disrupting the response-reward contingency critical for basal ganglia-driven procedural learning (Maddox et al., 2008). On the other hand, minimal feedback provides few cues for rule formulation, thus encouraging one to switch to the reflexive learning system relying on implicit stimulus-response associations (Chandrasekaran, Yi et al., 2014b). The key finding from Chandrasekaran, Yi et al. (2014b) shows that training the English listeners with minimal feedback *improved* their tone identification accuracy relative to using full feedback, suggesting that speech categories may be optimally learned by the reflexive system, at least for Mandarin lexical tones.

The current study aims to build on such findings by applying the COVIS/DLS framework to an instance of auditory categorization important for effective speech communication: talker identification. Here we examine the extent to which full or minimal feedback impacts the identification and learning of different talkers. We layer in an additional consideration that may interact with the informativeness of feedback: the extent to which the benefit of minimal feedback during talker identification may depend on whether the listener is familiar with the language or accent of the talker.

**Fig. 1:**
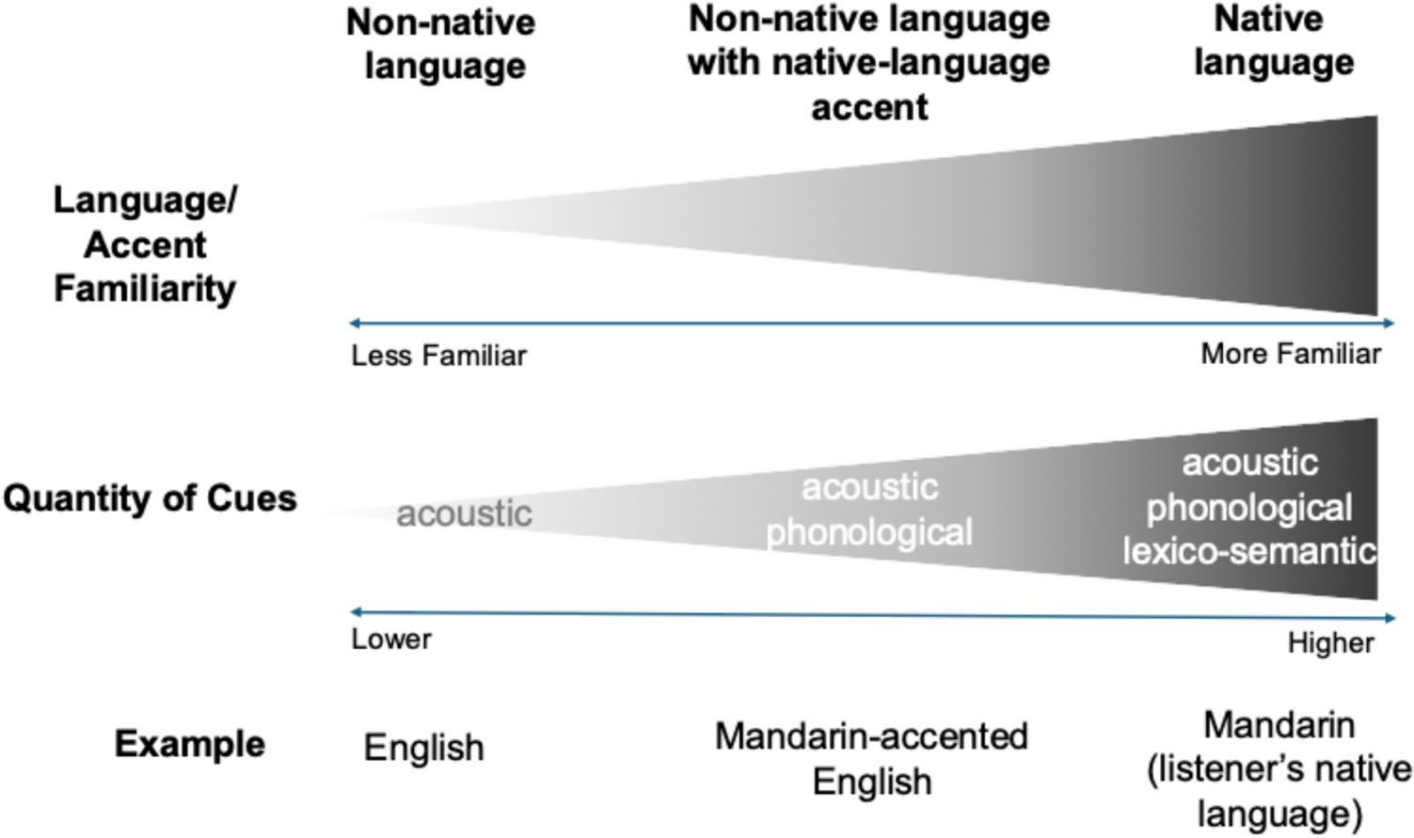
Illustration of the relationship between language/accent familiarity and the quantity of cues available in different language contexts. The top gradient bar represents the decreasing familiarity of language/accent from native language to nonnative language. The bottom gradient bar indicates the reduction in the quantity of cues available as the familiarity decreases

Prior work has consistently demonstrated a *language familiarity effect* (LFE), that is, listeners are more accurate at identifying voices in their native language compared to voices in an unfamiliar language (Goggin et al., 1991; Perrachione, 2018). Prior work has also provided evidence for the *other accent effect* (OAE; Stevenage et al., 2012)*,* with listeners finding it more difficult to identify talkers speaking their language in an unfamiliar or less familiar accent than talkers speaking in a more familiar accent (McLaughlin et al., 2019; Stevenage et al., 2012; Yu et al., 2021). These effects may occur because, as illustrated in Fig. 1, familiar language or accent allows listeners to use a wider range of available cues to decide who is talking, including not only acoustic cues to the talker’s physiological attributes but also linguistically relevant phonological and lexical-semantic cues (Levi, 2019; Mary Zarate et al., 2015; Xie & Myers, 2015). One prediction from the DLS framework would be that the abundance of cues in a familiar language or accent provides rich resources for explicit rule generation, creating a reflective-optimal learning condition. On the other hand, the fewer cues in an unfamiliar language or accent may provide a less sufficient basis for rule generation and encourage learning through the reflexive system. Therefore, if we expect that the benefit of minimal feedback over full feedback as found in tone category learning extends to talker learning, we may expect it to be greater in an unfamiliar^2^ language context (e.g., English speech for native Mandarin listeners).

Another consideration in examining talker learning is the decision dynamics underlying the categorization response. To evaluate learning success, one could focus exclusively on response accuracy, as is done in the majority of previous talker identification studies (e.g., Perrachione, 2018). Yet such measures reflect only the end results of identification and provide little insight into the underlying decisional processes, which are of theoretical interest considering the DLS model. As mentioned previously, the reflective system of the DLS model involves the generation of explicit rules. One behavioral consequence of this explicit rule processing may be that learners respond more slowly or cautiously due to the time and deliberation they put into checking the stimuli against the rules that are being held in working memory. Furthermore, it is possible that while response accuracy may be similar across different feedback or language/accent contexts, response times may differ. Thus, as described in detail below, we additionally analyze response time (RT) and apply a drift diffusion modeling (DDM; Ratcliff, 1978; Smith & Vickers, 1988; Myers et al., 2022) approach to examine the decision dynamics not captured by accuracy alone.

We employ DDMs to jointly model both accuracy and RT and better understand the decision processes related to categorization (Heffernan et al., 2021; McHaney et al., 2024; Paulon et al., 2021; Ratcliff, 1978; Roark et al., 2021, 2022; Smith & Vickers, 1988). Specifically, using recent advances in DDM that allow for assessing decision-making processes longitudinally over the course of learning (Paulon et al., 2021), we can examine how participants gather acoustic and/or linguistic information to make decisions about talker identity over time. DDMs assume that the subject makes decisions and initiates a response by stochastically accumulating evidence supporting one or more potential response options until the evidence meets a decision threshold (Ratcliff, 1978; Smith & Vickers, 1988). This process is modeled through two key parameters: evidence accumulation rate and decision threshold. Evidence accumulation rate is the rate at which evidence for a particular decision is gathered, with a faster rate indicating greater efficiency and higher quality of evidence extracted from stimuli (Ratcliff et al., 2016). Decision threshold represents how much evidence is required before a decision is made, with a higher threshold reflecting greater cautiousness and less efficiency, which can lead to slower responses (Bogacz et al., 2010; Ratcliff et al., 2016). Through these parameters, we examined whether responses were more efficient or cautious for some language or feedback conditions than others.

To this end, we designed a talker identification training experiment with feedback manipulation to examine how native Mandarin Chinese listeners make categorization decisions and learn to distinguish different talkers across three language contexts: 1) a Native Mandarin context, in which the talkers spoke in Mandarin, the listeners’ native language; 2) a Mandarin-accented English context, in which the talkers spoke English with a noticeable Mandarin accent; 3) a Native English context in which the talkers were native English speakers without a foreign accent. Given the well-established LFE and OAE, we expected identification performance, measured in accuracy and RT, to be highest for the Native Mandarin context, followed by the Mandarin-accented English context and then by the Native English context. Crucially, we hypothesized that compared with full feedback, minimal feedback targeting the reflexive system would result in higher identification accuracy, faster RT, faster evidence accumulation, and/or lower decision thresholds. We predicted these effects of feedback manipulation to be most obvious in the Native English context (where fewer cues are available to native Mandarin listeners) and least so in the Native Mandarin context, with the Mandarin-accented condition falling in the middle.

## Methods

### Participants

Seventy-nine native Mandarin Chinese listeners, 18–26 years old, with no history of language or hearing disorders, were recruited from the South China Normal University community. They were randomly assigned to complete the current study with either full feedback (*N* = 39, *M*_age_ = 20.7 years, *SD* = 2.18, 17 men/22 women) or minimal feedback (*N* = 40, *M*_age_ = 20.3 years, *SD* = 2.32, 17 men/23 women). All participants completed the experiment in person and received monetary compensation for their participation. Experimental procedures were approved by the South China Normal University Institutional Review Board and the Joint Chinese University of Hong Kong–New Territories East Cluster Clinical Research Ethics Committee. An additional participant in the full feedback condition did not complete all tasks and was excluded from further analyses. Participants completed the task in the Gorilla Experiment Builder (Anwyl-Irvine et al., 2020).

Prior to the learning tasks, participants completed demographics and language questionnaires and reported their familiarity and experience with different languages. Among all participants, around half reported knowing English (full: 20 out of 39; minimal: 24 out of 40) with an average self-reported proficiency of 4/10 (full: *M* = 4.10, *SD* = 1.74; minimal: *M* = 3.88, *SD* = 1.48). The number of participants knowing English did not differ significantly across the feedback conditions (χ^2^ = 0.306, *df* = 1, *p* = 0.58).

### Stimuli

**Fig. 2:**
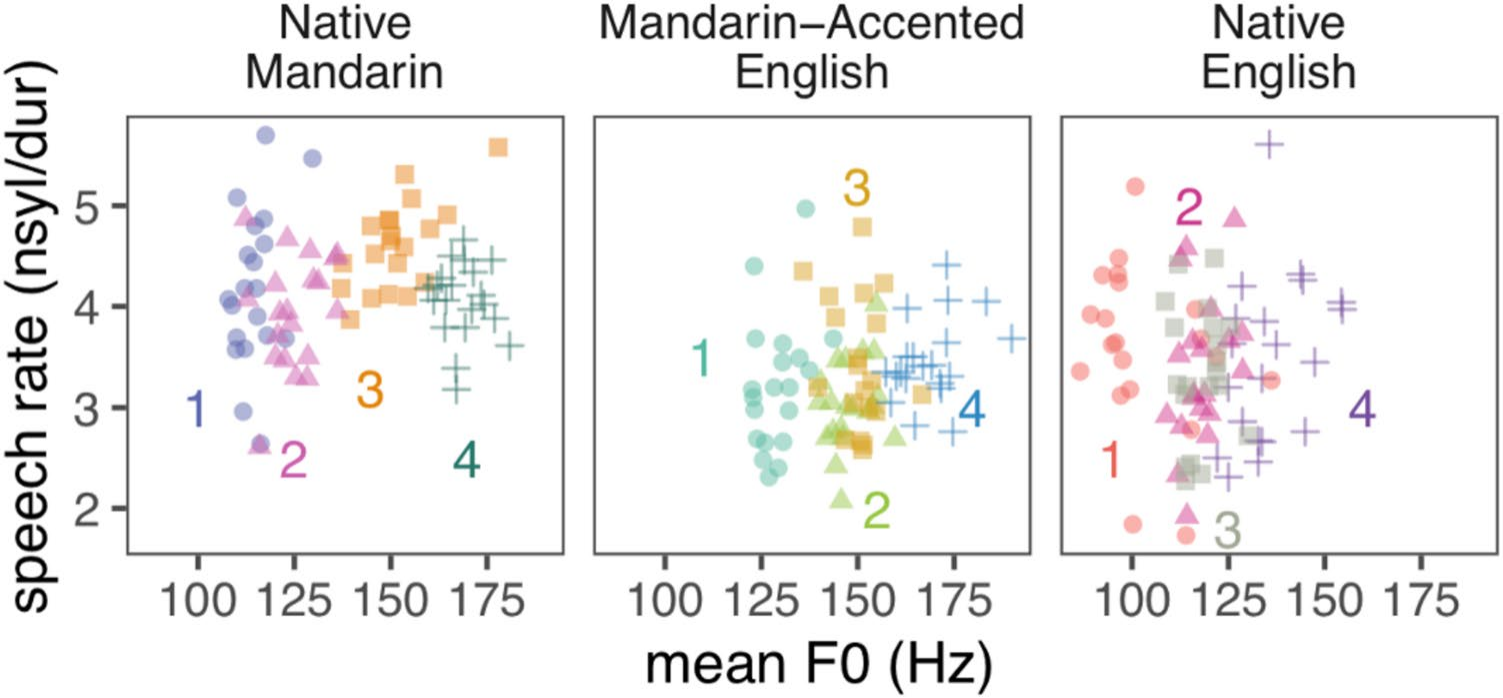
Distributions of all spoken sentences along the mean fundamental frequency (F0) and speech rate (number of syllables divided by total duration in seconds) dimensions. Each talker is shown in a different color and shape. (Color figure online)

Stimuli were short (1.0–2.6 s Hearing in Noise Test sentences (Soli & Wong, 2008) from the SpeechBox corpus (Bradlow, n.d.-b) and ALLSTAR corpus (Bradlow, n.d.-a). Four male speakers were selected from the corpus for each of the three language contexts (12 talkers total) following pilot testing with native Mandarin listeners, with the intent of selecting speakers that had no immediately clear idiosyncrasies that would make them stand out from other speakers in the same language context. In the Native Mandarin and Native English contexts, talkers were native speakers of either Mandarin Chinese (talker IDs: 05, 12, 32, 33) or American English (talker IDs: 53, 55, 61, 66), respectively, and produced sentences in their native languages. In the Mandarin-accented English context, talkers were native speakers of Mandarin Chinese and spoke sentences in English (talker IDs: 20, 21, 35, 39). All the talkers are young male adults 19–26 years old. As in natural speech, there were variations in acoustic features. Acoustic cues such as mean fundamental frequency (F0) and speech rate are reliable markers of talker identity and can support talker differentiation alone (LaRiviere, 1975; Lavner et al., 2001; Perrachione et al., 2019; Sambur, 1975; Skoog Waller et al., 2015; Van Dommelen, 1990). To illustrate the acoustic distance between voices, Fig. 2 visualizes how the stimuli naturally vary along these two commonly measured dimensions: mean F0 and speech rate (Bradlow & Pisoni, 1999; Johnson & Sjerps, 2021; Nolan, 1983; Winkler, 2007). For each language context, there were 10 training sentences and 10 test sentences. Each sentence was spoken by each of the four talkers in each language context for a total of 40 training sentences and 40 test sentences.

### Procedure

**Fig. 3:**
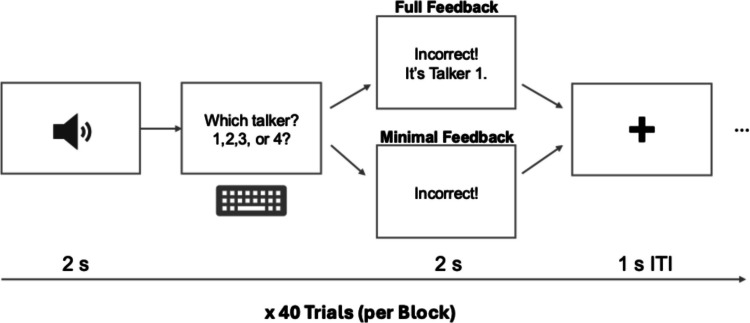
Procedure of the talker identification training. Participants heard a sentence for about 2 s and were prompted to identify who the talker was by selecting from four options. Depending on the feedback condition, they received either fully or minimally informative feedback. The intertrial interval (ISI) was 1 s. All instructions in the experiment were presented in written Chinese; the instructions shown in the figure have been translated into English

Participants wore Sennheiser HD280 Pro headphones and first completed a headphone screening to ensure they could hear the sounds in both ears (Milne et al., 2021). Afterwards, they were informed that their task was to learn to recognize the voices of new talkers. All participants completed all three language contexts with the different sets of speakers—Native Mandarin, Mandarin-accented English, and Native English. The order was counterbalanced across participants. At the beginning of each task, participants were told the language of the upcoming sentences (e.g., “In this task, you will hear sentences in English...”). On each trial of each task, participants heard a sentence randomly selected from the 40 sentences and identified the talker by selecting from four options (Fig. 3). In the full feedback condition, participants then received fully informative feedback (e.g., “Correct, it’s talker 1”; “Incorrect, it’s talker 2”) and in the minimal feedback condition, they received minimally informative feedback (e.g., “Correct”; “Incorrect”). Feedback was presented immediately after responses for 750 ms, and all participants were told to use the feedback to improve their performance. Feedback was followed by a 1-s intertrial interval. All instructions were presented in Chinese.

In each language context, participants completed five training blocks, where they heard each of the 40 sentences (10 sentences per talker) once. The final training block was followed by a generalization test, in which participants heard the 40 novel sentences spoken by the same four talkers (10 sentences per talker) and identified the talkers without any feedback. This helps assess listeners’ ability to transfer their learning from training to novel exemplars.

### Data analysis

#### Accuracy and response time

Response accuracy (a binary outcome: correct or incorrect) was analyzed using generalized linear mixed-effects regression (GLMER) models with a logit link function, implemented in the *lme4* package (Bates et al., 2015) of the open-source programming language R (R Development Core Team, 2024). Response times (RTs) were analyzed using linear mixed-effects models (LMER) within the same framework. To explore the effects of language context and feedback manipulation over the course of training, we first fitted a (G)LMER model with block, language context (reference level: Native English), feedback type (reference level: full), and all their interaction as fixed effects, including random intercepts for participants and sentences. An analysis of variance (ANOVA) test was then performed to assess the overall significance of the predictors. Next, we focused on two critical blocks that highlighted the extent of learning, the first and last (fifth) blocks and performed analyses separately in each of them. For response accuracy (either 1 or 0), a GLMER model was fitted including language context, feedback type, and all their interaction as fixed effects. The random effects included a by-participant random intercept, a by-sentence random intercept, and by-participant random slope for language context to account for intersubject and item-level variability. The mixed-effect model was also fitted to the data in the generalization test and the same analyses were repeated for RTs, which were modeled using the “lmer ()” function. *P* values of the fixed-effect terms were estimated using Satterthwaite’s approximation for denominator degrees of freedom from the *lmerTest* package (Kuznetsova et al., 2017).

#### Drift diffusion modeling

We employed the DDM (Paulon et al., 2021; Ratcliff, 1978; Roark et al., 2021, 2024; Smith & Vickers, 1988) to infer participants’ decision processes during talker learning in the three language contexts and two feedback conditions. Specifically, we examined the evidence accumulation (drift) rate and decision threshold (boundary) parameters as estimated by the DDMs fitted to the accuracy and RT data during the training blocks. We followed the approach used in Paulon et al. (2018), which allowed us to examine how the DDM parameters changed across learning blocks and differed across individuals in the 4AFC task. Implemented in the *lddmm* package in R (Paulon & Sarkar, 2024), the DDM developed by Paulon et al. (2021) fits an offset, drift rate, and boundary parameter for each combination of talker and response (e.g., Talker 1, response 1; Talker 1, response 2), each participant, and each 40-trial block. The offset parameter reflects the time taken by all nondecision processes (e.g., stimulus encoding, motor response). The drift rate parameter reflects the extraction of information from the stimulus relevant for the decision. The boundary parameter reflects the amount of evidence that would be collected before a decision is made. The DDM models were fitted with both correct and incorrect responses, but only the results from correct responses were analyzed to minimize error-induced variability and improve the accuracy of parameter estimates (Ratcliff & McKoon, 2008). In each combination of language context and feedback condition, we discarded trials with top 1% shortest or longest RTs as outliers and fitted a DDM using all talker-response combinations.

The *lddmm* package utilizes a Bayesian sampling approach implemented through Markov chain Monte Carlo simulations to estimate the posterior distributions of the DDM parameters. For the DDM model of each language context and feedback condition, we ran the simulations for 6,000 iterations with a burn-in of 2,000 iterations and a thinning factor of 5. The model estimated posterior distributions of the DDM parameters for each of the four talker response options. For simplicity, the parameter values were collapsed across the four responses at each posterior draw. We examined the initial and final training blocks and focused on drawing inferences about the effects of language context and feedback by computing posterior differences in evidence accumulation or decision threshold at each block. Specifically, we calculated the posterior differences between two levels of the language context or feedback factor and examined whether the 95% credible interval (CI) of the difference distribution excluded zero. Exclusion of zero from the 95% CI was considered evidence for statistical significance.

## Results

### Accuracy

**Fig. 4:**
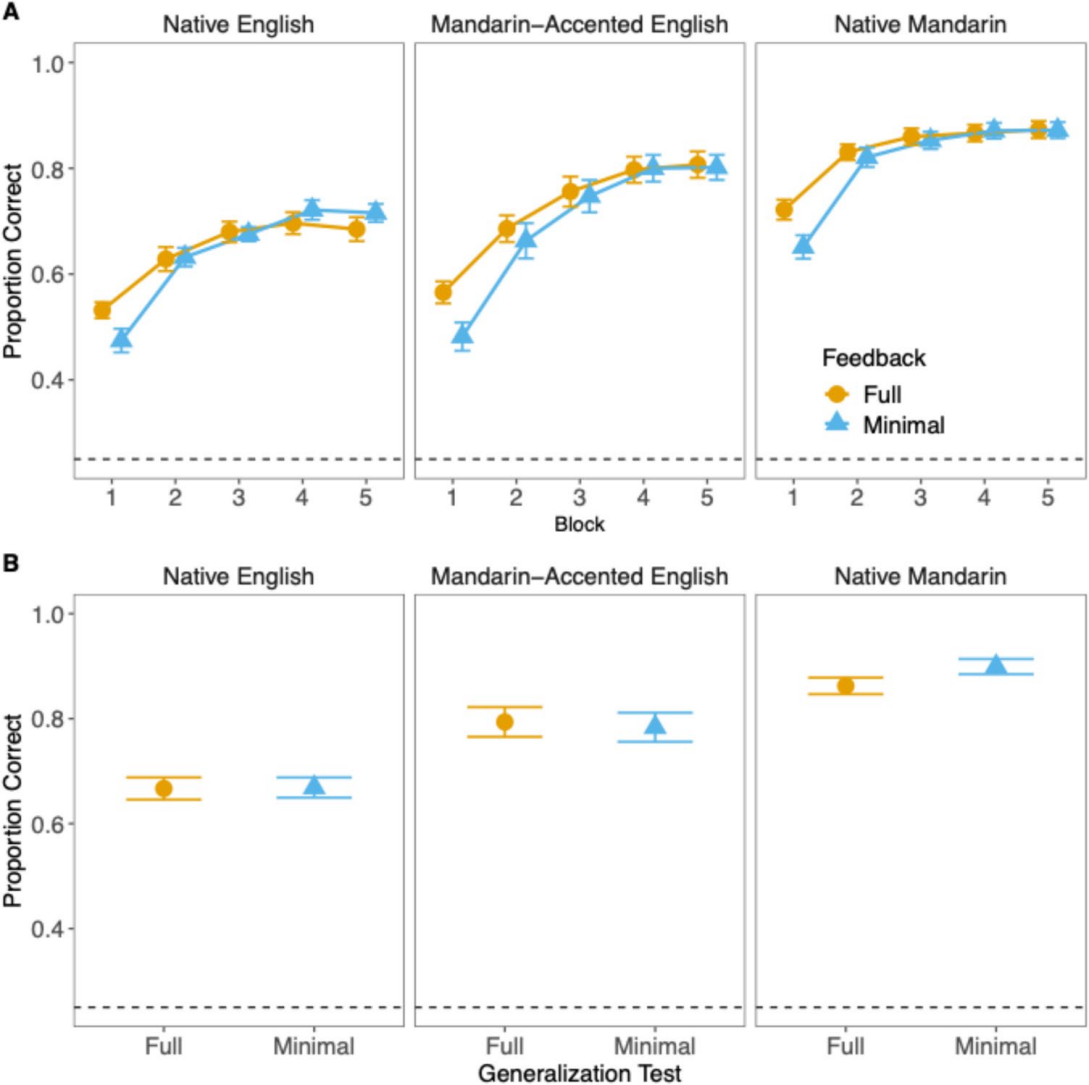
Mean identification accuracy (**A**) across training blocks 1 to 5 and (B) in the generalization test for all language contexts and feedback conditions. Error bars reflect one standard error*.* Dashed line indicates chance-level accuracy (0.25). (Color figure online)

Figure 4 shows the mean identification accuracy by feedback condition and language context over the training blocks and in the generalization test. Overall, identification accuracy increased monotonically over the course of training and was highest for the Native Mandarin context, followed by Mandarin-accented English and then by Native English. These trends were supported by the ANOVA test revealing significant main effects of block (χ^2^ = 132.627, *df* = 5, *p* < 0.001) and language context (χ^2^ = 131.111, *df* = 2, *p* < 0.001). There were also significant interactions between language context and block (χ^2^ = 39.486, *df* = 10, p < 0.001) and between feedback and block (χ^2^ = 16.205, *df* = 5, *p* = 0.006), suggesting that the effects of language context and feedback content varied across during the training.

To better understand the effects, we focused on block 1, block 5, and the generalization test and performed a mixed-effects analysis in each of them. The results are summarized in Table 1. In block 1, the accuracy of the Native Mandarin context, but not that of the Mandarin-accented English context, was significantly higher than that of the Native English context. Also, responses were more accurate in the full feedback condition than in the minimal feedback. Such a language context or feedback effect held across the levels of the other factor as the interaction terms were not significant. In Block 5, both Native Mandarin and Mandarin-accented English contexts showed higher accuracy compared with the Native English context, but there was no longer significant difference between the full and minimal feedback conditions. The same pattern of results was found in the generalization test, where listeners were presented with novel sentences from the same talkers that were not heard during training. Thus, at the initial stage of the training (Block 1), full feedback compared with minimal feedback resulted in more accurate identification; however, such benefit was no longer seen at the end of the training (Block 5) and in generalization to novel sentences.

**Table 1:** Summary output of the mixed-effect analyses of response accuracy in Block 1, Block 5, and generalization test

Block 1:				
	*β*	*SE*	*z*	*p*
Intercept (Native English, Full feedback)	0.124	0.081	1.527	0.118
Language context (Native Mandarin)	0.882	0.108	8.141	**< 0.001**
Language context (Mandarin-accented English)	0.153	0.105	1.447	0.148
Feedback (Minimal)	−0.228	0.111	−2.050	**0.040**
Feedback × Language context (Native Mandarin)	−0.115	0.151	−0.758	0.448
Feedback × Language context (Mandarin-accented English)	−0.128	0.145	−0.886	0.376
Block 5:				
Intercept (Native English, Full Feedback)	0.832	0.108	7.693	**< 0.001**
Language context (Native Mandarin)	1.325	0.137	9.658	**< 0.001**
Language context (Mandarin-accented English)	0.856	0.169	5.069	**< 0.001**
Feedback (Minimal)	0.135	0.136	0.993	0.321
Feedback × Language context (Native Mandarin)	−0.165	0.188	−0.880	0.379
Feedback × Language context (Mandarin-accented English)	−0.193	0.215	−0.898	0.369
Generalization test:				
Intercept (Native English, Full Feedback)	0.733	0.101	7.231	**< 0.001**
Language context (Native Mandarin)	1.290	0.154	8.388	**< 0.001**
Language context (Mandarin-accented English)	0.888	0.175	5.067	**< 0.001**
Feedback (Minimal)	0.004	0.132	0.034	0.973
Feedback × Language context (Native Mandarin)	0.411	0.220	1.862	0.063
Feedback × Language context (Mandarin-accented English)	−0.058	0.234	−0.247	0.805

### Response time

**Fig. 5:**
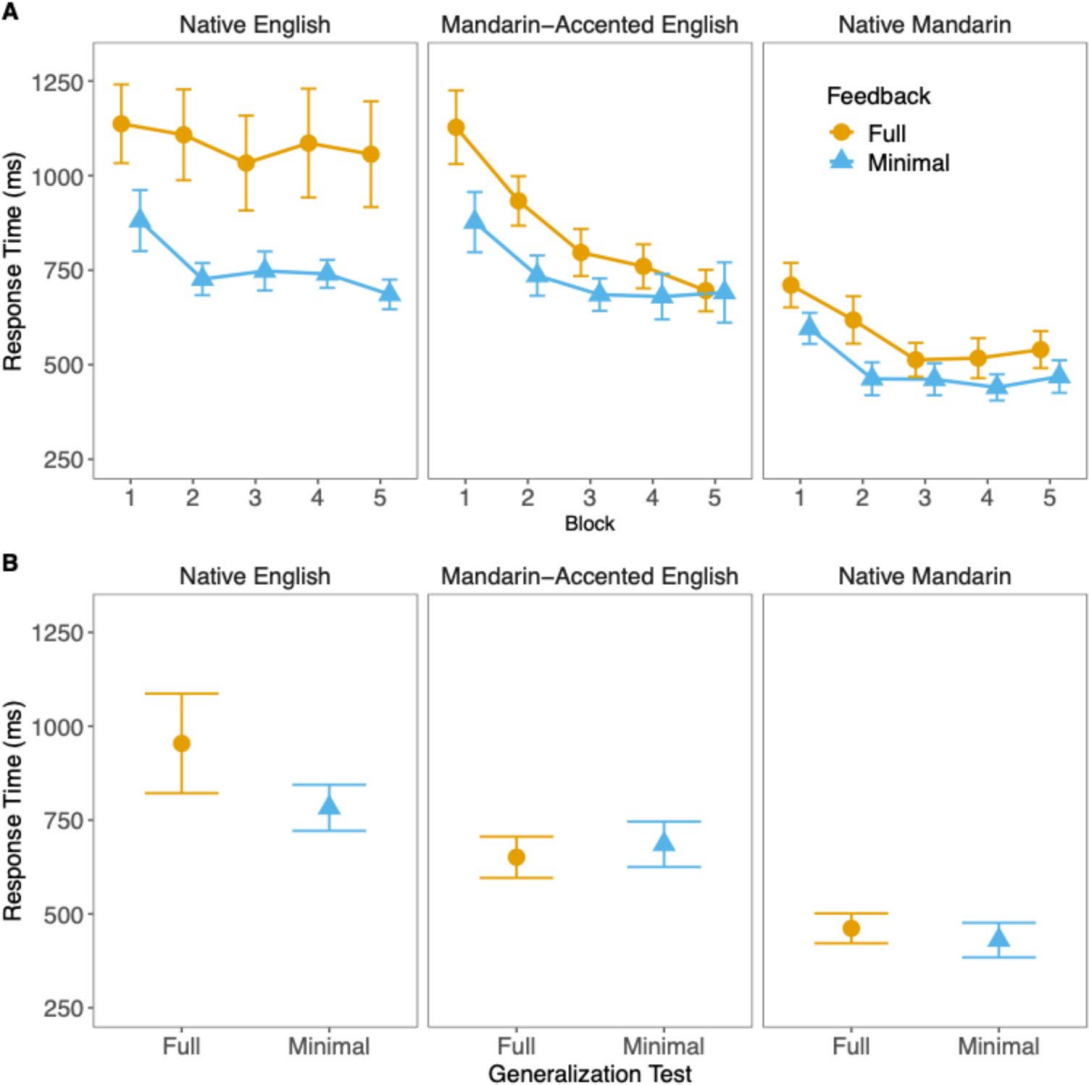
Mean identification reaction times (**A**) across training blocks 1 to 5 and (**B**) in the generalization test for all language contexts and feedback conditions. Error bars reflect one standard error*.* (Color figure online)

Figure 5 summarizes the RT data over the training blocks and in the generalization test. Consistent with the improvement in accuracy during the training, the participants’ responses generally became speedier over blocks, with the fastest responses in the Native Mandarin context, followed by Mandarin-accented English, and the slowest in Native English. These trends were suggested by significant main effects of block (χ^2^ = 164.0332, *df* = 5, *p* < 0.001) and language context (χ^2^ = 170.4565, *df* = 2, *p* < 0.001) according to the ANOVA test. Additionally, minimal feedback led to faster responses than full feedback (χ^2^ = 5.2151, *df* = 1, *p* = 0.02), though this effect varied across blocks (χ^2^ = 19.9034, *df =* 5, *p* = 0.001). There was also a significant interaction between block and language context (χ^2^ = 41.869, *df* = 10, *p* < 0.001), suggesting that RT improvements differed by language context throughout training.

Again, to better interpret the language context and feedback effects, we fitted separate mixed-effects models to training Blocks 1 and 5 and the generalization test, the results of which are summarized in Table 2. In Block 1, responses in the Native Mandarin context, but not those in the Mandarin-accented English context, were significantly faster than those in the Native English context. There was also a significant feedback effect indicating that responses were *faster* under the minimal feedback condition than the full feedback condition. This effect held across the three language contexts as the interactions were not significant. The same language context and feedback effects were observed in Block 5. Yet, this time the Mandarin-accented context also showed faster responses than the Native English context. Furthermore, there were significant interactions, which were driven by the fact that the reduction in RT of the minimal feedback condition occurred only in the Native English context (*t* = 2.610, *df* = 77.40, *p* = 0.033) but not in the other two contexts (Mandarin-accented English: *t* = 0.046, *df* = 78.00, *p* = 1.000; Native Mandarin: *t* = 1.101, *df* = 78.00, *p* = 0.823), as confirmed by post-hoc tests with *p* values Bonferroni-corrected for three comparisons. However, the patterns did not extend to the generalization test, where we only observed significant language context effects showing that familiar accents and languages resulted in faster identification responses.

**Table 2:** Summary output of the mixed-effect analyses of response time in Block 1, Block 5, and generalization test

Block 1:				
	*β*	*SE*	*t*	*p*
Intercept (Native English, Full Feedback)	1146.07	94.14	12.174	**< 0.001**
Language context (Native Mandarin)	−434.81	81.89	−5.309	**< 0.001**
Language context (Mandarin-accented English)	−17.77	101.68	−0.175	0.862
Feedback (Minimal)	−265.00	131.13	−2.021	**0.047**
Feedback × Language context (Native Mandarin)	150.61	115.14	1.308	0.195
Feedback × Language context (Mandarin-accented English)	14.27	140.63	0.101	0.920
Block 5:				
Intercept (Native English, Full Feedback)	1059.91	102.56	10.335	**< 0.001**
Language context (Native Mandarin)	−518.38	90.56	−5.724	**< 0.001**
Language context (Mandarin-accented English)	−363.47	101.88	−3.568	**< 0.001**
Feedback (Minimal)	−373.95	143.21	−2.611	**0.011**
Feedback × Language context (Native Mandarin)	301.96	127.29	2.372	**0.020**
Feedback × Language context (Mandarin-accented English)	369.45	141.07	2.619	**0.011**
Generalization test:				
Intercept (Native English, Full Feedback)	965.40	103.68	9.311	**< 0.001**
Language context (Native Mandarin)	−503.01	83.53	−6.022	**< 0.001**
Language context (Mandarin-accented English)	−313.22	100.96	−3.102	**0.003**
Feedback (Minimal)	−182.19	144.64	−1.260	0.212
Feedback × Language context (Native Mandarin)	151.63	117.48	1.291	0.201
Feedback × Language context (Mandarin-accented English)	217.03	139.24	1.559	0.123

The findings suggest that compared with full feedback, minimal feedback targeting the reflexive system initially resulted in faster talker identification across all contexts, despite lower accuracy. Interestingly, while accuracy across the two types of feedback was equivalent in the final training block, this reduction in response latency was found only for the Native English context, in which the talkers spoke in an unfamiliar language.

### Drift diffusion modeling

**Fig. 6:**
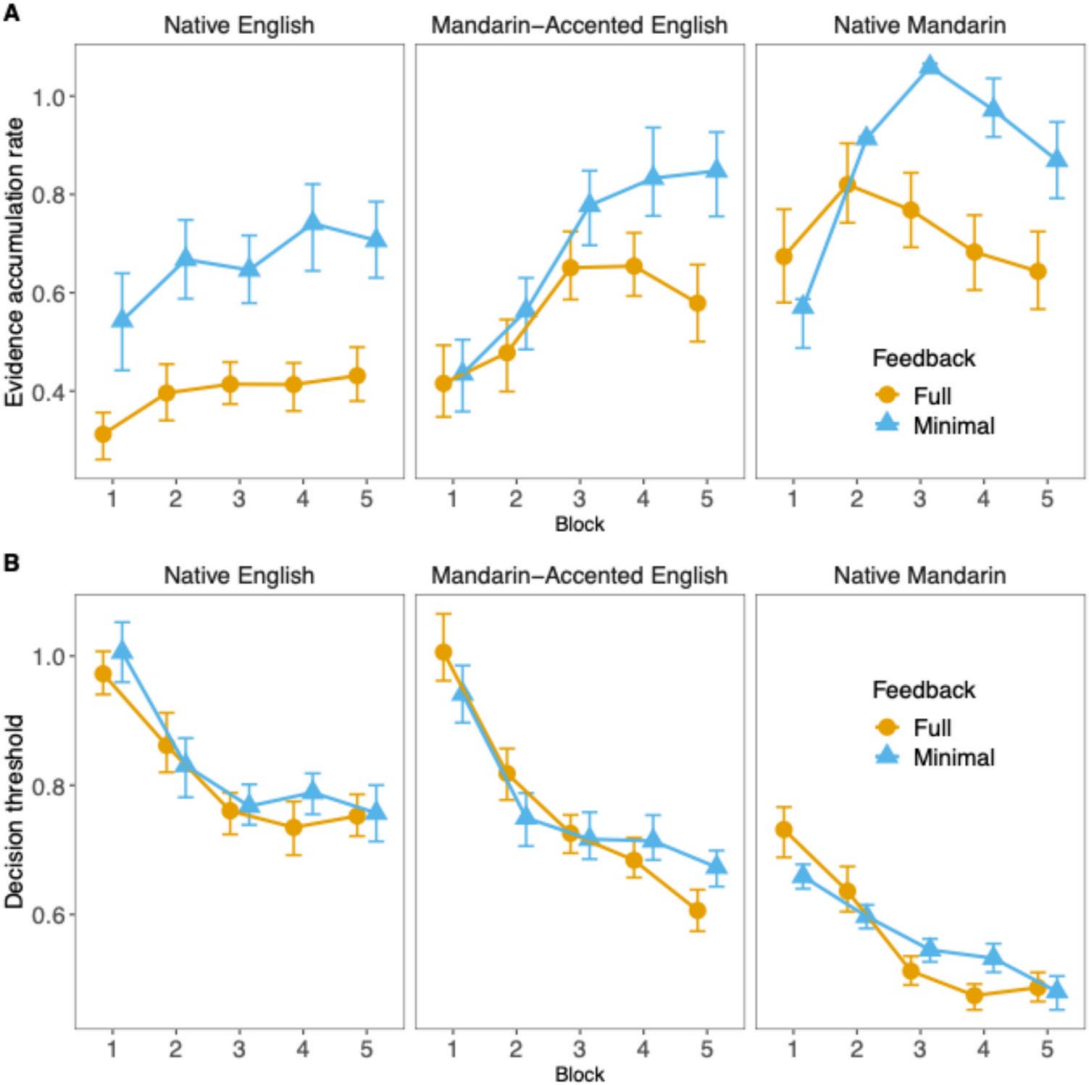
Drift diffusion model results. Estimated parameters for evidence accumulation rate (**A**) and decision threshold (**B**) for all language contexts and conditions. Error bars reflect 95% credible intervals*.* (Color figure online)

Figure 6 shows the mean and 95% CI of the posterior draws of the evidence accumulation and decision threshold parameters, divided by training block, language context, and feedback type. In general, evidence accumulation rates increased over blocks while decision thresholds became lower. There was an overall trend for the Native Mandarin context to show highest evidence accumulation rate and lowest decision thresholds, followed by the Mandarin-accented context and then by the Native English context. This trend, however, varied across blocks and feedback conditions. As we were interested in how feedback manipulation impacts talker identification learning, we concentrated on comparing full and minimal feedback in each language context and each stage during the training.

In Block 1, minimal feedback compared with full feedback was associated with higher faster accumulation for the Native English context only (95% CI [0.124, 0.352]). The difference in evidence accumulation between the feedback conditions was not significant for the Mandarin-accented English context (95% CI [−0.099, 0.127]) and Native Mandarin context (95% CI [−0.2133, 0.0003]). The decision threshold was not significantly different across the two feedback types in the Native English context (95% CI [−0.024, 0.09]) but was lower in the minimal feedback condition for the Mandarin-accented English context (95% CI [−0.143, −0.002]) and in the Native Mandarin context (95% CI [−0.109, −0.023]). Thus, initially during the training, talker identification was more efficient with faster evidence accumulation and no reduction in decision threshold when the participants learned to identify English-speaking talkers with minimal feedback. No such feedback effects were observed for the other two language contexts.

In Block 5, minimal feedback relative to full feedback led to higher evidence accumulation rates across all three language contexts (Native English: 95% CI [0.183, 0.365]; Mandarin-accented English: 95% CI [0.166, 0.380]; Native Mandarin: 95% CI [0.122, 0.342]). Decisions thresholds in general did not differ across feedback conditions (Native English: 95% CI [−0.05, 0.051]; Native Mandarin: 95% CI [−0.047, 0.027]) except they were higher for minimal feedback than full feedback in the Mandarin-accented English context (95% CI [0.028, 0.108]). The results indicated that talker training with minimally informative feedback compared with full feedback ultimately resulted in more efficient identification responses regardless of the accent or language of the talkers.

## Discussion

Motivated by the COVIS and DLS framework, the current study examined the extent to which content of feedback impacts the learning of new talkers in different language contexts. Regardless of the language context or feedback type (full or minimal), participants’ identification responses became more accurate and faster with training, with increased evidence accumulation rates and reduced decision boundaries during training. Importantly, while full feedback initially led to higher accuracy in early training blocks, this advantage (full > minimal) did not extend to later blocks or the generalization test. Interestingly, response times and evidence accumulation rates were generally faster across all language contexts in the minimal feedback condition than the full feedback condition, suggesting that minimal feedback facilitated more efficient decision-making. Minimal feedback facilitates talker learning with a higher quality of information extracted from stimuli in all language contexts. Overall, these findings demonstrate that minimal feedback targeting at the reflexive system facilitates category decision making, especially as learners progress beyond the initial training stages.

### Effect of familiar language/accent on talker learning

Aligning with prior studies (e.g., Goggin et al., 1991; Levi, 2019; Perrachione, 2018; Thompson, 1987), we found higher accuracy and faster RTs in the Native Mandarin context compared with the Native English context, replicating the LFE. Our DDM analysis further indicated that when identifying a talker speaking one’s native language, decision processes are supported by faster evidence accumulation and lower decision thresholds. Consequently, cues crucial for talker identification may be more readily available and of higher quality, facilitating judgments with less information than in nonnative language contexts.

Our findings also extended previous research on the OAE (Stevenage et al., 2012), which has predominantly focused on talkers speaking the listener’s native language but with different accents. By including the Mandarin-accented English context, we found that at least in the later stages of learning and generalization test, native Mandarin speakers could identify individuals speaking English with a Mandarin accent faster and more accurately those speaking English with a native American English accent. Such a finding suggests that listeners may be able to access native-language phonological cues in an unfamiliar language, consistent with prior research showing improved identification in talkers producing pseudowords that have similar sound patterns of their native language compared with listening to foreign speech (Mary Zarate et al., 2015; Perrachione et al., 2015; Xie & Myers, 2015), OAE is not limited to languages that the listener knows; rather, familiar sound patterns may be exploited to aid talker identification regardless of the lexical-semantic content of the speech.

### Role of minimal feedback in promoting efficient talker learning

As for the effect of feedback content, we observed a shift in the relative impact of the two feedback types across training blocks, with minimal feedback showing a stronger effect in enhancing talker learning in response efficiency during the later stages of training. Initially, listeners’ identification accuracy across the three language contexts was slightly higher with full feedback than with minimal feedback for initial learning, which was consistent with the finding that full feedback showed a benefit over minimal feedback when categories can be differentiated based on verbalizable rules (Dunn et al., 2012; Maddox et al., 2008; Yi & Chandrasekaran, 2016). This suggests that at least at the very initial stage, learning is primarily mediated by a rule-based reflective learning system, in which learners use explicit rules to identify verbalizable differences between talkers and rapidly develop identification strategies. However, the advantage for full feedback did not persist. Throughout the rest of training, accuracy in both the full and minimal feedback conditions converged, and in the generalization test, which assesses the transfer of learning to novel exemplars and evaluates learning robustness, accuracy in the full feedback condition was no longer superior. These results add to emerging evidence that suggests that even for reflexive-optimal categories, initial learning may be driven by a more reflective learning mechanism. Presumably, this allows for the system to develop some level of positive feedback, enough for the transfer of control to the reflexive system. The observed shift in the effect of minimal feedback is further supported by prior neuroimaging and computational modeling data highlighting a switch from early reflective learning to more reflexive and procedural-based learning, which relates to reduced demands on working memory and executive attention and higher accuracy in decision making.

Furthermore, at later training blocks, the evidence accumulation rate was generally higher for minimal feedback than full feedback, indicating more efficient decision making while maintaining accuracy. Minimal feedback may result in a reduction of the cognitive load and minimizes interference, allowing learners to focus on the most important features without being overwhelmed by irrelevant features. Also, minimal feedback may encourage learners to integrate multidimensional cues, fostering a more sensory-driven representation associated with information-integration reflexive learning rather than rule-based reflective learning (Feng et al., 2019). Therefore, minimal feedback, which promotes learning through the reflexive, procedural-based mechanism (Chandrasekaran, Yi et al., 2014b; Maddox et al., 2008; Yi & Chandrasekaran, 2016), ultimately leads to greater efficiency in talker identification.

### Enhancing talker learning in an unfamiliar language through minimal feedback

It must be noted that the improved efficiency with minimal feedback is particularly prominent in the Native English context, as indicated by a significant interaction between Language context and Feedback in the final training block, driven by the fact that the reduction in RT of the minimal feedback condition occurred only in the Native English context (see Fig. 5A). Native Mandarin speakers showed faster response times with minimal feedback particularly in the Native English context without compromising accuracy, as accuracy remained similar between the two feedback conditions throughout the training beyond the first block. Such greater efficiency was further reflected in the evidence accumulation rate, which, as previously mentioned, was higher for minimal feedback than for full feedback at later training blocks, indicating more efficient decision-making while maintaining accuracy. One possible explanation for this effect is that when processing talkers in an unfamiliar language context, where there are fewer cues accessible (as illustrated in Fig. 1), excessive reflective processing may hinder the transfer of control to reflexive learning. By providing only response correctness, minimal feedback enables learners to consolidate the most salient features of the voices while focusing on the most critical cues. This helps them extract relevant patterns more effectively without being overwhelmed by excessive information, thereby reducing cognitive load and facilitating more efficient learning and quicker adaptation to new talkers.

The RT advantage for minimal feedback in the English context did not reach statistical significance in the generalization test (see Fig. 5B), where novel stimuli were introduced. One possible explanation is that the introduction of novel sentence stimuli in the generalization test prompted the listeners to engage in explicit processing of the stimuli and fall back on the reflective mechanism to some extent. The lack of statistical significance may reflect a temporary reengagement of the reflective system due to attention to novel aspects of the stimuli. However, as shown in Fig. 5B, the Native English context still showed a trend toward faster RTs for minimal feedback than full feedback, indicating potential transfer of training effects to generalization. We speculate that with extended exposure to the generalization stimuli, the trend may become significant. This remains an open empirical question worth exploring in future research.

The current findings provide a number of implications and directions for future research. First, the fact that the benefit of minimal feedback relative to full feedback was manifested primarily as greater decision efficiency highlights the need to consider measures other than accuracy, which was the sole metric for evaluating responses in most previous talker identification research (for reviews, see Levi, 2019; Perrachione, 2018). Including RT and DDM could reveal subtle effects of experimental conditions and participants’ strategies in the decision-making process (e.g., whether they are being more efficient or not). Furthermore, our DDM results suggest that changes in processing efficiency during talker identification may be captured primarily by one DDM measure but not both. While the evidence accumulation rate showed consistent effects, decision threshold results were more variable—for example, the mean decision threshold was higher for minimal feedback than for full feedback for Mandarin-accented English at Block 5. Further research is needed to explore why the decision threshold findings were less consistent and what implications this may have for talker identification. Third, we also observed that faster responses with minimal feedback compared to full feedback was especially evident in the Native English context. Such an observation raises the possibility that learning to identify talkers is more likely to recruit the reflexive learning mechanism in the context of unfamiliar language/accent compared with familiar language/accent. If this is the case, learners would be expected to rely less on verbalizable talker cues in a nonnative language or accent, since too much reflective processing on verbalizable and explicit cues may hinder procedural-based learning.

Fourth, the current findings should be interpreted with respect to the specific listener population (native Mandarin speakers) and stimuli used (isolated sentences). Further research is needed to assess whether the observed learning patterns generalize to other listener populations, different language pairs, and more naturalistic speech materials, such as spontaneous or conversational speech. While English is described as a “nonnative” and “unfamiliar” language here, our participants were university students with some degree of English exposure. An interesting direction of future research would be to examine languages with which Mandarin speakers in general have minimal or no prior exposure (e.g., Estonian or Navajo). For these cases, we predict that patterns similar to those found here in the English context (e.g., faster RTs in minimal feedback than full feedback condition) will likely emerge, but may be more pronounced or robust. Investigating a wider variety of linguistic contexts and speech types will provide greater insight into how language familiarity and speech naturalness together influence talker identification and learning strategies. Fifth, future research could examine how individual differences, such as working memory capacity or attentional control, interact with task demands to shape the balance between reflexive and reflective learning strategies. Finally, it is unknown whether our participants indeed exploited different sets of cues across the three language contexts while listening to the sentence. It is possible that while listeners generally use acoustic cues such as F0, they do so more or less or focus on a particular dimension (e.g., F0 height or movement) depending on the language context. An important avenue of future work will be to address this question by, for example, assessing the extent to which the brain tracks acoustic and linguistic features of the stimuli in different conditions (e.g., Brodbeck et al., 2022; Di Liberto et al., 2015; Gillis et al., 2022).

To conclude, our study situates the talker identification problem within the DLS framework of category learning. The results provide evidence for the advantage of minimal feedback over full feedback, which was reflected primarily as faster identification speed and higher evidence accumulation rates especially in an unfamiliar language context. This advantage suggests that similar to learning to identify speech sound categories (Chandrasekaran, Yi et al., 2014b), learning to identify talkers may be better facilitated in conditions that encourage the reflexive system. We also replicated the well-documented LFE and OAE. Our findings have implications for real-world cross-linguistic communication, where listeners often need to identify and adapt to unfamiliar voices across varying languages and accents. For example, such challenge arises in multilingual educational settings, where teachers and students may need to quickly adapt to unfamiliar speech pattern in linguistically diverse classrooms; in international customer service, where representatives interact with callers from varied linguistic backgrounds; and in aviation communication, where air traffic controllers coordinate with pilots from around the world. Improved understanding of how language familiarity shapes talker identification can support more effective communication in these domains and inform the design of speech technologies, such as speaker recognition systems and voice authentication tools that rely on accurate speaker differentiation across diverse linguistic profiles. It is recommended that future work incorporate measures that capture response efficiency and decision dynamics, such as response time analyses and drift diffusion modeling, to more precisely characterize the cognitive mechanisms underlying talker identification. Moreover, continued efforts are needed to examine the extent to which listeners rely on specific acoustic or linguistic cues in different language contexts. Addressing such questions will be critical for advancing our understanding of how language experience modulates talker identification processes.

## Data Availability

The data and materials associated with this study can be obtained online (https://osf.io/g7r9q/).

## Footnotes

1 We acknowledge that although terms like ‘native’ and ‘non-native’ are widely used in psycholinguistics, they have been critiqued for being vague, exclusionary, and overly simplistic (Brown et al., 2023; Cheng et al., 2021). Here we use them as descriptive, operational labels to distinguish languages which our participants presumably had distinct experiences with. Specifically, ‘native’ refers to languages acquired (or individuals acquiring them) in early childhood (typically before age 6) through immersive exposure, and ‘nonnative’ refers to languages learned (or individuals learning them) after early childhood primarily through formal instruction.

2 Note that the term ‘unfamiliar’ (or ‘familiar’) is used here as a convenient label to indicate that English, relative to Mandarin, is likely less familiar to our participants due to their limited proficiency and reduced day-to-day exposure. The term is therefore used in a relative sense and does not imply that English is entirely unknown to them.

## Code availability

Coded in R, which is publicly available online (https://osf.io/g7r9q/).
